# Uncovering the spread of drug-resistant bacteria through next-generation sequencing based surveillance: transmission of extended-spectrum β-lactamase-producing Enterobacterales by a contaminated duodenoscope

**DOI:** 10.1186/s13756-024-01386-5

**Published:** 2024-03-08

**Authors:** Cansu Cimen, Erik Bathoorn, Arjo J. Loeve, Monika Fliss, Matthijs S. Berends, Wouter B. Nagengast, Axel Hamprecht, Andreas Voss, Mariëtte Lokate

**Affiliations:** 1 Department of Medical Microbiology and Infection Prevention, University of Groningen, University Medical Center Groningen, Hanzeplein 1, 9700RB Groningen, The Netherlands; 2 Institute for Medical Microbiology and Virology, University of Oldenburg, Oldenburg, Germany; 3 Department of Biomechanical Engineering, Faculty of Mechanical Engineering, Delft University of Technology, Delft, The Netherlands; 4 Certe Medical Diagnostics and Advice Foundation, Department of Medical Epidemiology, Groningen, The Netherlands; 5 Department of Gastroenterology, University of Groningen, University Medical Centre Groningen, Groningen, The Netherlands

**Keywords:** Endoscopic retrograde cholangiopancreatography (ERCP), Duodenoscope, Nosocomial transmission, Contamination, Extended-spectrum β-lactamase (ESBL), *Klebsiella pneumoniae*, *Citrobacter freundii*, Next-generation sequencing (NGS), Multi locus sequence typing (MLST), SHV-12, CTXM-15

## Abstract

Contamination of duodenoscopes is a significant concern due to the transmission of multidrug-resistant organisms (MDROs) among patients who undergo endoscopic retrograde cholangiopancreatography (ERCP), resulting in outbreaks worldwide. In July 2020, it was determined that three different patients, all had undergone ERCP with the same duodenoscope, were infected. Two patients were infected with *bla*_CTX-M-15_ encoding *Citrobacter freundii*, one experiencing a bloodstream infection and the other a urinary tract infection, while another patient had a bloodstream infection caused by *bla*_SHV-12_ encoding *Klebsiella pneumoniae*. Molecular characterization of isolates was available as every ESBL-producing isolate undergoes Next-Generation Sequencing (NGS) for comprehensive genomic analysis in our center. After withdrawing the suspected duodenoscope, we initiated comprehensive epidemiological research, encompassing case investigations, along with a thorough duodenoscope investigation. Screening of patients who had undergone ERCP with the implicated duodenoscope, as well as a selection of hospitalized patients who had ERCP with a different duodenoscope during the outbreak period, led to the discovery of three additional cases of colonization in addition to the three infections initially detected. No microorganisms were detected in eight routine culture samples retrieved from the suspected duodenoscope. Only after destructive dismantling of the duodenoscope, the forceps elevator was found to be positive for *bla*_SHV-12_ encoding *K. pneumoniae* which was identical to the isolates detected in three patients. This study highlights the importance of using NGS to monitor the transmission of MDROs and demonstrates that standard cultures may fail to detect contaminated medical equipment such as duodenoscopes.

**Supplementary Information:**

The online version contains supplementary material available at 10.1186/s13756-024-01386-5.

## Background

Transmission of multidrug-resistant organisms (MDROs) by contaminated duodenoscopes is a recurring and significant complication of endoscopic retrograde cholangiopancreatography (ERCP) [1]. Flexible endoscopes are a primary concern regarding transmission of microorganisms related to reusable medical devices [1]. Several outbreaks have been reported worldwide because of the use of contaminated duodenoscopes, often involving multidrug-resistant Gram-negative bacteria particularly those from the taxonomic order of Enterobacterales, such as *Citrobacter freundii* and *Klebsiella pneumoniae* [2–5]. A meta-analysis reported a contamination rate of 15.3% for reprocessed patient ready ERCPs, indicating that contamination of ERCP duodenoscopes may not be rare [6].

Regular microbiological surveillance by means of culturing contaminating bacteria is recommended to monitor reprocessing and prevent contamination of duodenoscopes and patient-to-patient cross-transmission of microorganisms [7, 8]. However, even when standard duodenoscope cultures showed no growth, several outbreaks of Gram-negative MDRO following ERCP have been reported, highlighting the limitation of routine culture-based methods in identifying these microorganisms [4, 9–12]. Also, establishing epidemiological connections between bacterial isolates solely through phenotyping (i.e., culturing) is challenging, due to its limited resolution in differentiating closely related strains. Therefore, additional measures are necessary to ensure patient safety and prevent transmission of MDROs, such as the use of advanced diagnostic techniques including genomic sequencing and analysis, owing to their capacity for precise MDRO identification [13, 14].

Next-generation sequencing (NGS) has emerged as a transformative tool in hospital settings with providing a swift and precise identification and monitoring microorganisms [15]. The routine application of NGS in clinical microbiology has demonstrated significant advantages in outbreak detection and management as sequencing the entire genome of microorganisms allows for comprehensive genetic analysis, providing insights into the relatedness of strains [16]. The capability of NGS to facilitate the rapid characterization of outbreak strains, enabling healthcare facilities to implement timely and targeted infection control measures [17]. Furthermore, the utilization of NGS for genotyping can identify the source of infection by revealing whether the analyzed microorganisms form genotypic clusters, particularly in the context of healthcare-associated infections [18]. Although the uptake of the technology is still far from universal, it has been shown that the implementation of routine use of NGS for MDRO typing in hospital settings can significantly lower healthcare costs [18, 19].

For this reason, we aimed to demonstrate the effectiveness of routine-based NGS in uncovering the source of an outbreak, by pinpointing a contaminated ERCP duodenoscope as the origin of Extended-spectrum β-lactamase (ESBL)-producing *Citrobacter freundii* and *Klebsiella pneumoniae* in patients. This study highlights the role of advanced genomic methods in enhancing infection control in healthcare settings.

## Methods

### Study setting

The University Medical Center Groningen (UMCG) is a 1400-bed tertiary academic center in the north of the Netherlands. Approximately 500 ERCPs are performed every year at the Endoscopy Center of the department of Gastroenterology and Hepatology. To perform ERCP, Olympus Model TJF-Q180V duodenoscopes were in use throughout the outbreak period.

### Case settings and definitions

**Fig. 1:**
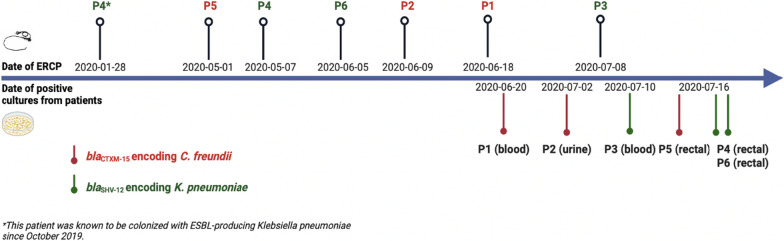
Timeline of ERCP procedure and infection and colonization of patients. (*This patient was known to be colonized with bla_SHV-12_ K. pneumoniae since October 2019; ERCP, endoscopic retrograde cholangiopancreatography; ESBL, Extended-spectrum β-lactamase; P1, patient 1; P2, patient 2; P3, patient 3; P4, patient 4; P5, patient 5; P6, patient 6). Created with Biorender

ESBL-producing isolates are sequenced using NGS, typed, and stored at the department of Medical Microbiology and Infection Prevention in our center. In July 2020, two closely related *bla*_CTX-M-15_ encoding* C. freundii* isolates were detected: one from a pediatric patient (*patient 1*) causing a bloodstream infection (BSI) and the other from an adult patient (*patient 2*) in the gastroenterology department causing a urinary tract infection (Fig. 1). Patient records showed that both patients had undergone ERCP with the same duodenoscope (duodenoscope 294) nine days apart in June 2020. In July, another patient (*patient 3*) from the gastroenterology ward developed a BSI due to *bla*_SHV-12_ encoding *K. pneumoniae* two days after undergoing ERCP with the same duodenoscope.

The following case definitions were used: *probable cases* are patients with ESBL-producing isolates that cluster with isolates from other patients who had undergone ERCP with the same scope, and *proven cases* showed isolates that also cluster with isolates detected on the scope.

### Epidemiological investigations

Following the detection of ESBL-producing *C. freundii* and *K. pneumoniae* in patients who underwent ERCP with duodenoscope 294, the scope was withdrawn, and ambi-directional epidemiological investigations were started. This investigation included identification of all potential exposed patients (probable and proven cases) and intensive investigation of the suspected duodenoscope.

#### Case investigations

Patients were listed who underwent ERCP with duodenoscope 294 from 2020-01-23 (last negative surveillance culture of the duodenoscope) to the day the duodenoscope was withdrawn from the endoscopy center. The hospital records of these patients, including their microbiological test results were examined to assess the prevalence and patterns of ESBL-producing microorganism related infections/colonization within the facility.

Additionally, throat and rectal swabs were obtained using ESwab® (COPAN Diagnostics, CA, USA) from patients (n = 39) who had undergone ERCP with duodenoscope 294 and were subsequently discharged from the hospital. Patients were provided with explicit instructions for self-sampling, with a focus on prompt shipment and strict adherence to temperature conditions (4 °C to 25 °C) during transport. To eliminate the potential for contamination from an alternative source, a selection of hospitalized patients who underwent ERCP with another duodenoscope during this period were also screened. Selective agar (Mediaproducts BV, Groningen, the Netherlands) was used for screening on Extended-spectrum β-lactamase producing Enterobacterales (ESBL-E) and were examined after both 24 and 48 h.

In addition, we checked retrospectively whether the strains circulating during the outbreak differed from strains that had been detected in our hospital between 2018 and 2020. This was done by analyzing the genomic data of all 21 ESBL-producing *C. freundii* isolates and all 75 K*. pneumoniae* isolates.

#### Duodenoscope investigation

Duodenoscope 294, which was introduced in our center in March 2019, was withdrawn from clinical use in July 2020 because of the suspicion as the source of the outbreak. Its last surveillance culture was performed in January 2020 and was negative.

Antegrade and retrograde culture samples were taken from the duodenoscope on five different days between 2020-07-10 and 2020-07-28, using nationally advised sampling methods [20]. Three additional cultures were obtained from the duodenoscope on different days (2020-08-04, 2020-08-06, 2020-08-10) with the addition of sampling the scope’s forceps elevator while moving it and using gauzes in addition to the swabs. On 2020-08-10, the interior of the duodenoscope was examined using a fiberscope.

**Fig. 2:**
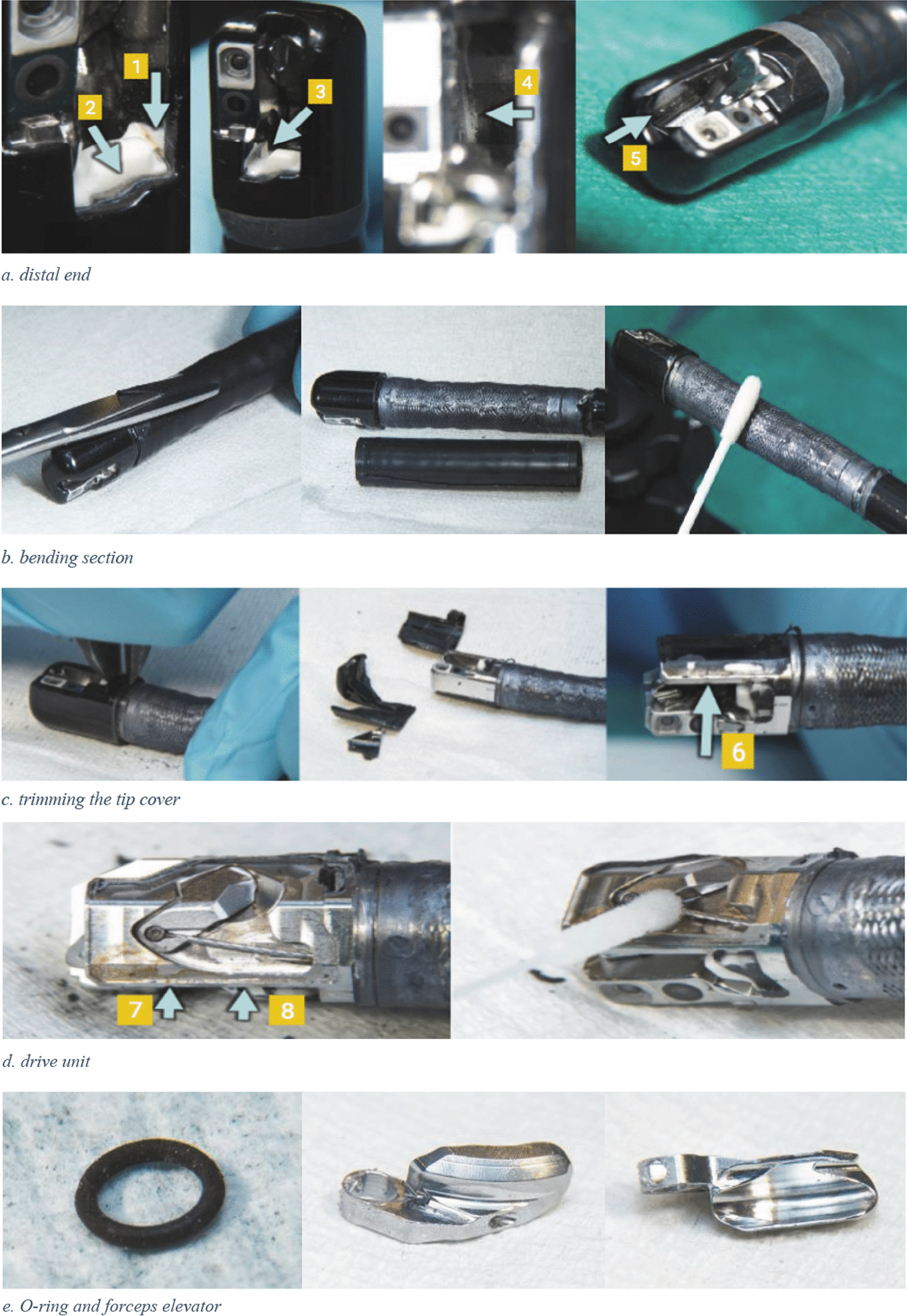

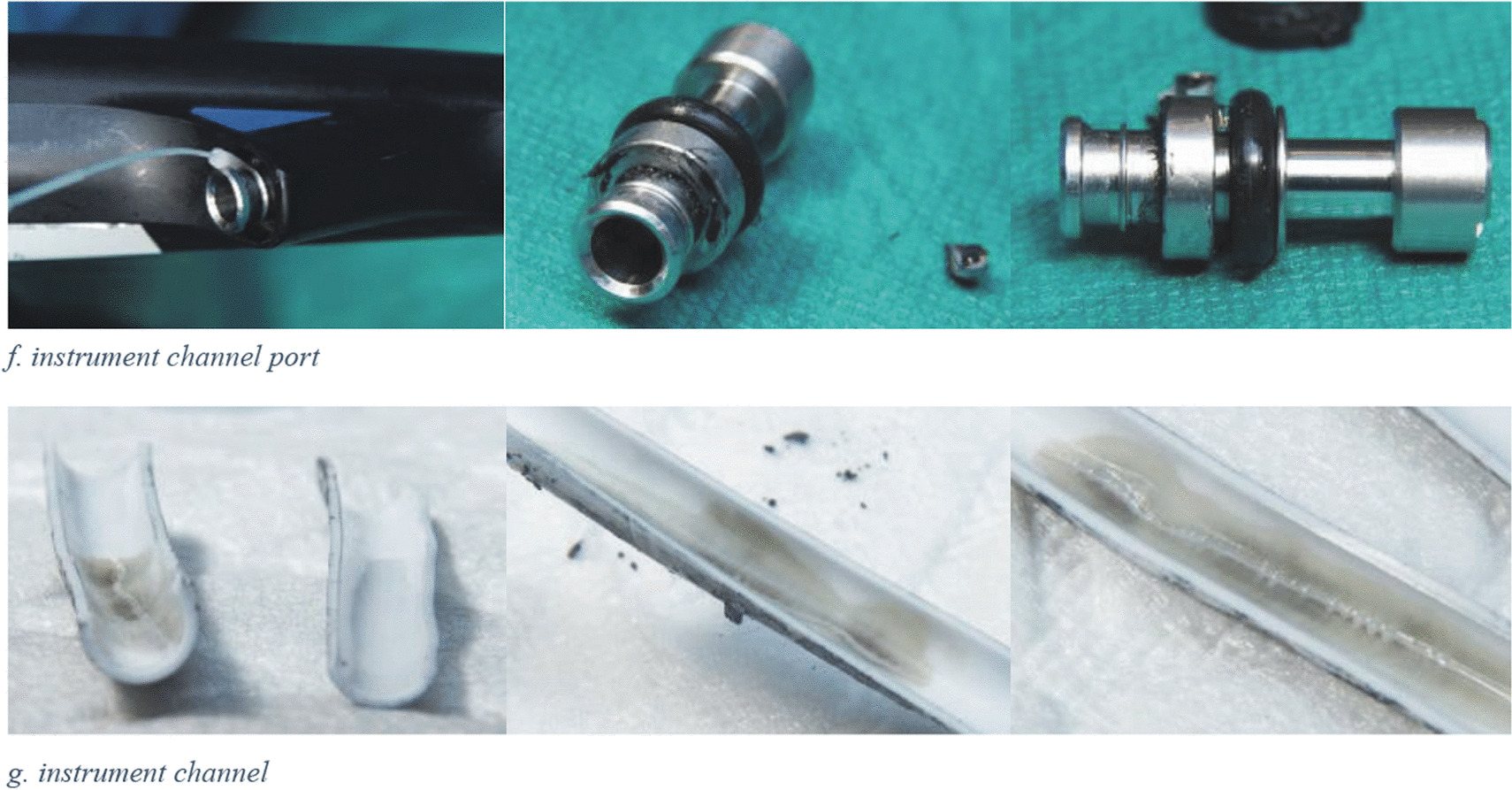
Dismantling and sampling the ERCP duodenoscope. a, red-brown residue in groove (1) on the forceps elevator drive unit side and whitish-grey residue in the border above the white block around the instrument channel exit (2); white–gray residue (3) in groove on camera side; white deposit (4) on the bottom under the forceps elevator; white crystals (5) next to and below the forceps elevator; b, cutting and removing the cardan rubber, sampling the exposed surface; c, trimming the tip cover and the possible break (6) in the cementing around the arm cover.; d, brownish-red deposit and/or discoloration on the frame of the drive unit, with an apparent intrusion trace (7), corresponding to the previously observed possible interruption of cementing (8); f, sampling the instrument channel port; removed instrument channel port viewed from the entrance and port from the side; g, left half the piece of instrument channel with the brownish-yellow area; the ribbed scratch mark shows a kink where there is also a kink or dent in the tubing; Yellow discoloration of the tubing material with grey cloudy discoloration on the inner wall

On 2020-12-10 a team of experts consisting of a medical devices’ technician, an endoscope technician, a microbiologist from Olympus, and an infection preventionist conducted a thorough destructive investigation on duodenoscope 294 (Fig. 2). The investigation was lead, documented, and photographed by an independent external investigator from the University of Delft, the Netherlands. The team diligently followed the instructions of the independent investigator, ensuring the preservation of the investigation’s independence. All relevant parts and channels were sampled under clean conditions for microbiological research (Fig. 2).

### Sequencing and data analysis

Bacterial isolates obtained through a routine diagnostic procedure as described before including environmental screening was grown overnight on blood agar plates at 37 °C [21]. The DNeasy UltraClean Microbial Kit (QIAGEN, Hilden, Germany) was used to extract the genomic DNA following the manufacturer’s instructions. Libraries preparation was performed using Nextera XT v2 kit (Illumina, San Diego, CA, USA), and was followed by sequencing on the MiSeq platform (Illumina). Trimming and de novo assemblies were performed in CLC Genomics Workbench v21 (QIAGEN, Hilden, Germany). The quality trimming setting were default except for quality limit (0.01). De novo assemblies were done with default setting and word-size 29. For quality metrics the following parameters were used: number of contigs < 1,000; N50 > 15,000; maximum contig length > 50,000; percentage reads used for assembly > 90%; coverage > 30x; percentage of suspected genome length > 90%-115%. Ridom SeqSphere + v6.0.2 (Ridom GmbH, Münster, Germany) was used for Multi Locus Sequence Typing (MLST) according to public databases for molecular typing and microbial genome diversity (PubMLST) schemas and core genome MLST (cgMLST) with a species-specific ad-hoc schemas. For *K. pneumoniae* ad-hoc schema contained 4891 different loci and for *C. freundii* scheme containing 4632. The cgMLST scheme was developed using the seed genome *K. pneumoniae* subsp. pneumoniae NTUH-K2044 (GenBank: NC_012731.1). In case of *C. freundi* ad-hoc schema was created using the seed genome *C. freundii* CFNIH1 (GenBank: CP007557.1). Presence of known resistance genes was performed using the online tool ResFinder (https://cge.food.dtu.dk/services/ResFinder/).

All sequence data (including two *C. freundii* isolates submitted for a separate project (PRJEB44899)) have been submitted to European Nucleotide Archive (ENA) under the study accession number PRJEB67940 and the dataset is available in Additional file 1.

## Results

### Case investigations

Reviewing the patient records revealed that one patient (*patient 4*) had been colonized with *bla*_SHV-12_ encoding *K. pneumoniae* since October 2019 and underwent ERCP with duodenoscope 294 on 2020-01-28 and 2020-05-07 respectively (Fig. 1).

**Fig. 3:**
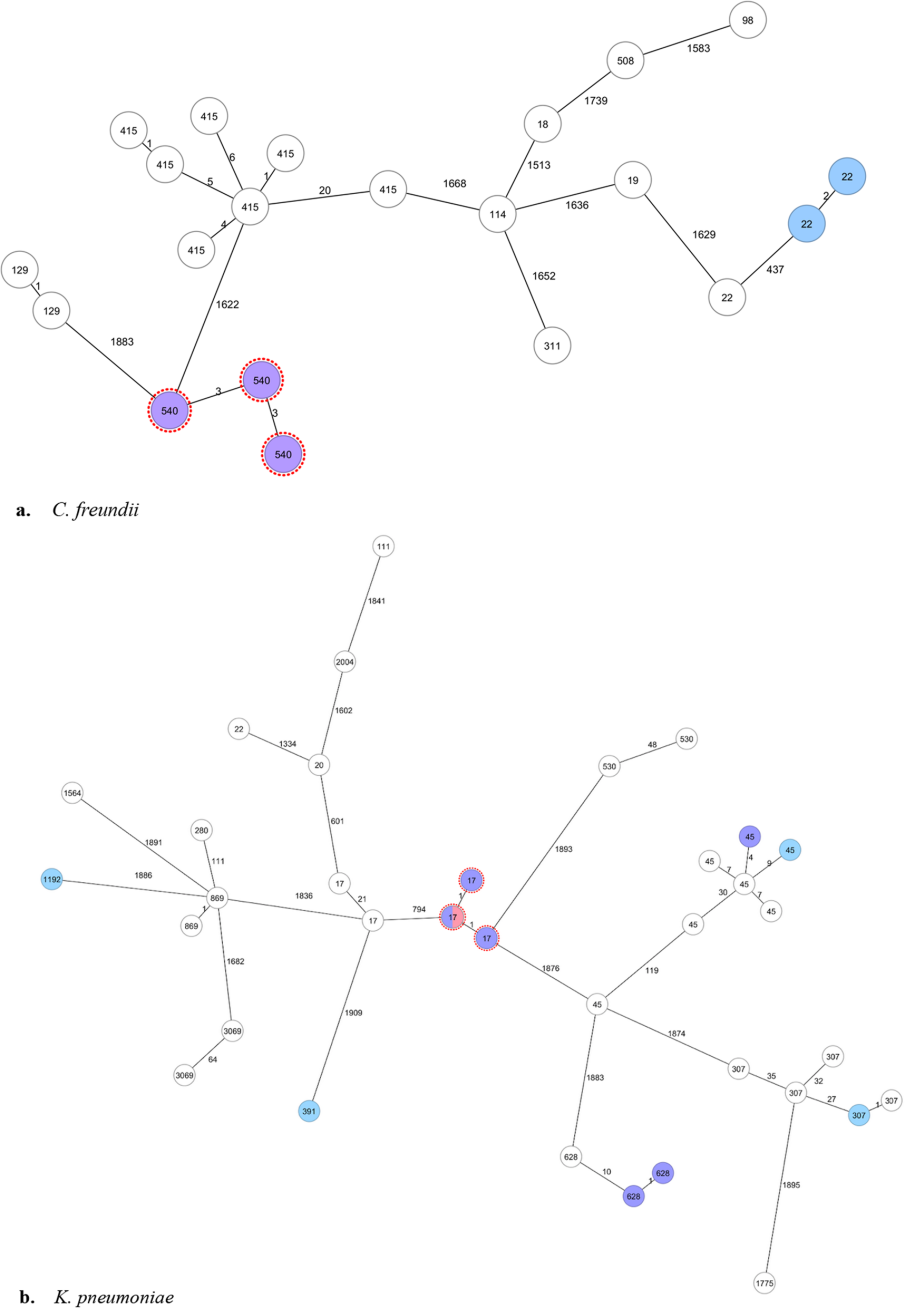
Overview of ESBL-producing *C. freundii* and *K. pneumoniae* isolates with ST assigned by cgMLST analysis performed at UMCG during 2018–2020, highlighting different isolate categories and outbreak strains. Strains were selected corresponding to the same lineages. Blue, isolates obtained from patients who underwent endoscopy before the first positive culture; purple, isolates obtained from patients who underwent ERCP before the first positive culture; pink, isolates obtained from the duodenoscope; red dots around the purple-colored isolates, outbreak strains of *C. freundii* (ST540) and *K. pneumoniae* (ST17). **a**, ST22 C. freundii were obtained from the same patient at different times; **b**, ST628 K. pneumoniae strains were identified in two patients who shared a room, and both underwent ERCP procedures with different duodenoscopes in October 2019

Out of the 39 patients who were screened, 37 returned the requested swab, reflecting a compliance rate of 95%. Within these 37 patients, three additional patients with ESBLs were found matching the initial cases by cgMLST: one with *bla*_CTX-M-15_ encoding* C. freundii* (*patient 5*), and two with *bla*_SHV-12_ encoding* K. pneumoniae* (*patient 4* and *patient 6*) (Fig. 3).

Six cases in total were identified comprising three *probable* cases and three *proven* cases.

Eight ERCP duodenoscopes were in use between 2018-01-01 and 2020-12-3. Notably, our additional case finding method revealed no other strains related to the described outbreak were found in patients who underwent ERCP with duodenoscopes other than the suspected duodenoscope (Fig. 3, Additional file 1).

#### Duodenoscope investigation

**Fig. 4:**
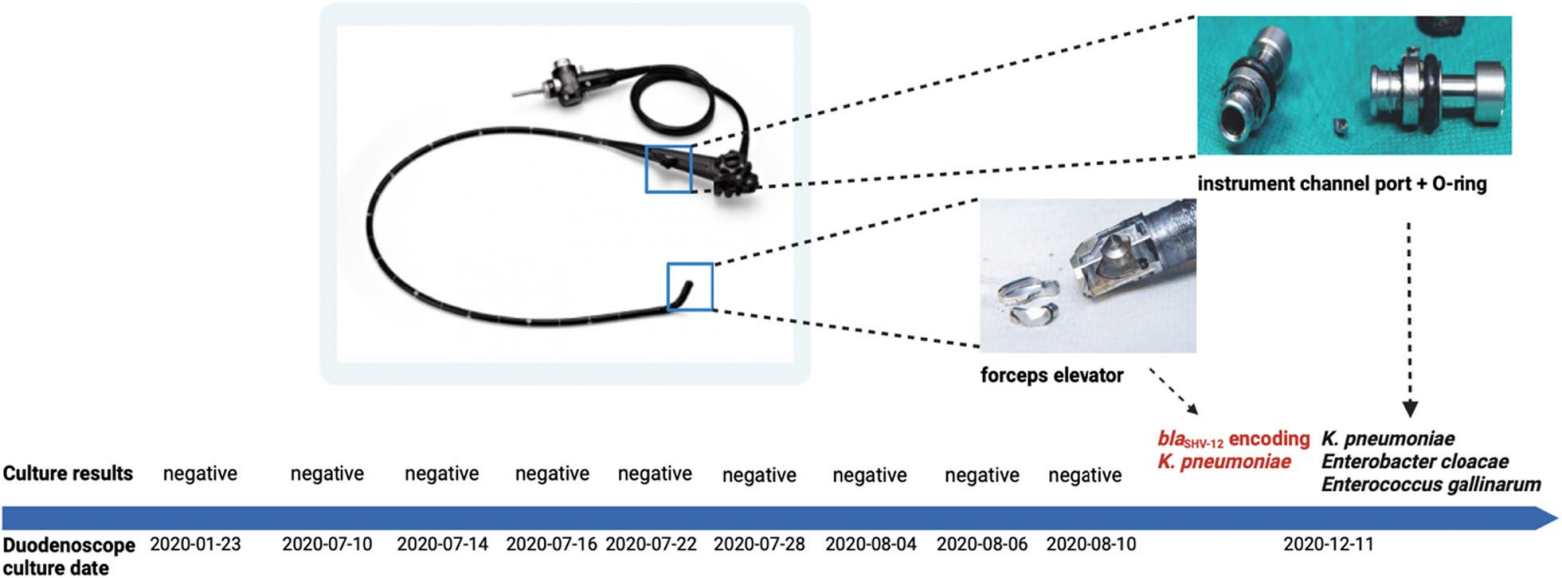
Culture results of the ERCP duodenoscope

Five culture tests using standard methods (antegrade and retrograde culture samples) and an additional three tests using other methods described above did not yield any detection of microorganisms in the suspected duodenoscope. Samples taken from the forceps elevator during dismantling were positive for *bla*_SHV-12_ encoding *K. pneumoniae* (Fig. 4). The strain was subjected to NGS and considered to be identical to the strain detected in *patients 3, 4* and *6* (*proven* cases). In addition, the instrument channel port (Fig. 2f) including the O-ring (Fig. 2e) were found to be contaminated by a wild type *K. pneumoniae*, *Enterobacter cloacae,* and *Enterococcus gallinarum*.

The additional samples taken with a brush from the instrument channel (Fig. 2g), which exhibited major damage and signs of biofilm formation as observed through the fiberscope, did not show any growth of microorganisms.

## Discussion and conclusion

Our study underscores the role of routine NGS-based surveillance and accompanying extensive epidemiological investigation in the detection and management of MDRO outbreaks, as demonstrated by the identification of a contaminated ERCP duodenoscope in this incident. Despite intensive consecutive cultures of the duodenoscope failing to detect any microorganisms, guiding NGS analysis and dismantling of the duodenoscope revealed that ESBL-producing *K. pneumoniae* was transmitted by the suspected duodenoscope. The results of the study indicate that *patient 4*, a carrier of ESBL-producing *K. pneumoniae* since October 2019, probably contaminated the duodenoscope during ERCP in May 2020. However, the retrospective nature of the study limits our ability to precisely determine the exact time of transmission of the microorganism.

Although the ESBL-producing *C. freundii* strain was not detected on the suspected duodenoscope, it was likely responsible for transmission in three patients from different wards. This conclusion is supported by the strain’s presence in the patients who underwent ERCP with the same duodenoscope, displaying a clear epidemiological link. Notably, no instances of the same *C. freundii* strain have been detected among patients over the past three years except for these three *probable* cases, according to the NGS database. The delayed dismantling and sampling of the duodenoscope may have contributed to the negative culture result for *C. freundii*, as bacterial survival in an inanimate environment decreases over time.

Over the last decade, an increasing number of hospital-associated infections and outbreaks worldwide linked to contaminated duodenoscopes have been reported [6, 22, 23]. This increase may be attributed to infections due to MDROs gaining attention and being a driving force in resource-seeking [24]. In our case, it was noteworthy to encounter a new ESBL-E variant that caused infection in two patients. Since certain MDROs are subjected to molecular typing via NGS and stored in our center, we were able to retrospectively trace the source of the phylogenetically identical isolates. The absence of any detected microorganisms in the duodenoscope through consecutive intensified cultures may suggest that duodenoscope-associated infections (DAIs) can be easily underestimated, despite the previously mentioned rise in their occurrence over the past decade. Our investigation shows that sequencing plays a key role in identifying pathogen transmissions and preventing outbreaks beyond routine microbiological diagnosis.

The application of NGS in investigating outbreaks has proven invaluable for numerous bacterial pathogens, offering critical insights into outbreak definition, transmission networks, and epidemiological aspects [25–28]. Genetic data obtained through NGS not only identify unexpected modes of transmission but also have the potential to interrupt silent transmission chains [28]. Additionally, genomic sequencing plays a crucial role in establishing direct links between patients and environmental or infrastructure isolates, enhancing our understanding of the outbreak’s scope within the hospital [29]. The significance of our center’s approach lies in the comprehensive sequencing, typing, and storage of clinically important multidrug-resistant organisms, including ESBL-producing isolates, ensuring a robust and enduring database for enhanced epidemiological insights and outbreak detection. We acknowledge that the widespread adoption of these methods in all clinical laboratories is not realistic, given considerations of technical feasibility and the capacity to invest in molecular testing equipment [26]. Yet, individual laboratories play a crucial role by preserving unusual bacterial isolates and engaging in collaborative efforts with reference laboratories for further sequencing. While it is a common practice in clinical laboratories to discard of bacterial isolates once test results are reported, their retention becomes pivotal during outbreaks, facilitating retrospective epidemiological research and providing access to genetic material, as presented in this study.

Transmission of microorganisms by contaminated duodenoscopes is mainly attributed to inadequate reprocessing procedures, which can be due to various reasons [6]. One of the reasons is human factors, such as personnel adherence to infection control practices and to manufacturer’s manuals during reprocessing [30]. The Food and Drug Administration (FDA) ‘Human Factors Studies’ has shown that there is a low level of compliance to the reprocessing guidelines among the personnel responsible for reprocessing, and that they mainly failed to adequately clean the movable parts [31]. Another reason is the complex design of the flexible ERCP duodenoscopes with multiple channels and narrow lumens, which can cause difficulties in thoroughly disinfecting them entirely before storage [7, 23, 32, 33]. For instance, in 2015 the FDA sent a warning letter to Olympus Corporation regarding the newly designed TJF-Q180V type duodenoscope due to reported DAIs via this model [34]. This model was introduced as having a closed channel system as the elevator wire channel was blocked by an O-ring and a fixed cap at the distal end that could hinder adequate cleaning of the forceps elevator. An outbreak report described this complex system of the duodenoscope as resulting in inadequate reprocessing [35]. It was argued that although the original purpose of the O-ring was to avoid the need to clean the elevator wire channel, it did not achieve the desired success. The O-ring was even claimed to increase the risk of contamination due to leakage being overlooked because the space behind the O-ring is inaccessible during routine surveillance sampling [35]. Subsequently, Olympus Corporation declared the need for modification to the device design due to aforementioned consequences and recalled the duodenoscopes from endoscopy centers in 2016 [36]. The TJF-Q180V model duodenoscopes currently in use in our center were among those that were recalled and remodeled before getting back to the clinic. Dismantling of the duodenoscope in our center revealed that the forceps elevator, O-ring, and instrument channel port were contaminated. Our findings confirm the difficulty of reprocessing this intricately designed duodenoscope, as well as how these contamination locations can be missed during routine culture. Disposable elevator cap (DEC) duodenoscopes offer a solution to reprocessing challenges posed by the complexity of traditional duodenoscopes [37]. In a recent clinical trial, DEC duodenoscopes showed decreased contamination post-high-level disinfection compared to standard scopes, though persistent contamination, primarily in the channel rather than the elevator region, remained a concern [38].

Several guidelines recommend routine microbiological testing of duodenoscopes to detect inappropriate reprocessing [8, 20, 32]. In our department, reprocessed duodenoscopes undergo rigorous surveillance culturing every two weeks, adhering to national guidelines for enhanced infection prevention practices. However, there is currently no universal protocol for the sampling method [1]. Many cleaning methods have been described for the complex design of flexible duodenoscopes, including the swab-rinse method for the biopsy channel port, the flush/brush/flush method for the channel and the flush-through method for the channel lumens [39]. In addition to these specific methods, antegrade and retrograde sampling methods, which are named based on the end of the duodenoscope from which the rinse water is collected, are also described [40]. A surveillance protocol based on retrograde sampling was developed in our center and retrograde sampling was found to be more effective and sensitive than antegrade sampling in duodenoscope surveillance [40]. During the implementation of the aforementioned protocol, our center detected an outbreak caused by a multidrug resistant *Pseudomonas aeruginosa* in 2009. Although missed in routine surveillance cultures, detection of the specific duodenoscope responsible for the outbreak was again performed using retrograde sampling during the investigation [5]. In our case, microorganism detection could not be achieved without disassembling the duodenoscope despite successive surveillance cultures, including antegrade and retrograde sampling. Our findings highlight the need to review existing protocols and to reach a consensus on how duodenoscopes can be sampled most effectively.

There are limitations to the study. Primarily, the analysis of probable transmission samples and retrospective samples was contingent upon availability, which may not comprehensively represent all potential cases. Additionally, the time lapse between the initial detection of MDRO and subsequent screening poses a challenge that individuals might no longer be colonized with the transmission strain, potentially leading to an underestimation of the identified cases, thereby no longer harboring the transmission strain at the time of screening. This time gap could lead to an underestimation of the total number of cases associated with the outbreak, potentially skewing the understanding of its scope and impact. Lastly, as this report is based on a single-center experience, there is a lack of systematic assessment or meta-analysis regarding the cost-effectiveness of routine typing for ESBLs.

In conclusion, this report underscores the significance of using NGS to monitor MDROs and for uncovering the transmission of MDROs via contaminated medical devices; despite intensive cultures, the transmission of ESBL-producing microorganisms was only revealed through NGS analysis and dismantling of the duodenoscope. For this reason, our study also emphasizes the challenges in detecting and preventing hospital-acquired infections caused by contaminated duodenoscopes. Dismantling the suspected duodenoscope and showing the existence of ESBL-producing *K. pneumoniae* highlights the fact that ERCP duodenoscopes could be a source of transmission of MDROs despite negative surveillance cultures. These findings underscore the importance of molecular typing and sequencing in identifying the source of infections and preventing outbreaks. Hence, these results call for a review of existing protocols and a consensus on improved sampling methods to enhance the detection and prevention of DAIs, while also emphasizing the necessity for future research to systematically assess the cost-effectiveness of routine NGS typing.

### Supplementary Information

**Additional file 1**. Supplementary sequence data for the study isolates.

## Data Availability

The datasets used or analyzed for the study were provided within the manuscript and additional data can be requested from the corresponding author.

## Abbreviations

cgMLST: Core genome MLST
DAIs: Duodenoscope associated infections
ENA: European nucleotide archive
ERCP: Endoscopic retrograde cholangiopancreatography
ESBL: Extended-spectrum β-lactamase
ESBL-E: Extended-spectrum β-lactamase producing Enterobacterales
FDA: The food and drug administration
MDROs: Multidrug-resistant organisms
MLST: Multi locus sequence typing
NGS: Next-generation sequencing
PubMLST: Public databases for molecular typing and microbial genome diversity
UMCG: University Medical Center Groningen
